# Activation of Glutathione Peroxidase 4 as a Novel Anti-inflammatory Strategy

**DOI:** 10.3389/fphar.2018.01120

**Published:** 2018-10-03

**Authors:** Cong Li, Xiaobing Deng, Xiaowen Xie, Ying Liu, José Pedro Friedmann Angeli, Luhua Lai

**Affiliations:** ^1^BNLMS, State Key Laboratory for Structural Chemistry of Unstable and Stable Species, College of Chemistry and Molecular Engineering, Peking University, Beijing, China; ^2^Peking–Tsinghua Center for Life Sciences, Peking University, Beijing, China; ^3^Center for Quantitative Biology, Peking University, Beijing, China; ^4^Rudolf Virchow Center for Experimental Biomedicine, University of Würzburg, Würzburg, Germany

**Keywords:** arachidonic acid metabolic network, GPX4, enzyme activator, allosterism, drug discovery, anti-inflammatory, ferroptosis

## Abstract

The anti-oxidative enzyme, glutathione peroxidase 4 (GPX4), helps to promote inflammation resolution by eliminating oxidative species produced by the arachidonic acid (AA) metabolic network. Up-regulating its activity has been proposed as a promising strategy for inflammation intervention. In the present study, we aimed to study the effect of GPX4 activator on the AA metabolic network and inflammation related pathways. Using combined computational and experimental screen, we identified a novel compound that can activate the enzyme activity of GPX4 by more than two folds. We further assessed its potential in a series of cellular assays where GPX4 was demonstrated to play a regulatory role. We are able to show that GPX4 activation suppressed inflammatory conditions such as oxidation of AA and NF-κB pathway activation. We further demonstrated that this GPX4 activator can decrease the intracellular ROS level and suppress ferroptosis. Our study suggests that GPX4 activators can be developed as anti-inflammatory or cyto-protective agent in lipid-peroxidation-mediated diseases.

## Introduction

Glutathione peroxidase 4 (GPX4; E.C.: 1.11.1.12), also called phospholipid hydroperoxide glutathione peroxidase (PHGPx), is a selenium-dependent glutathione peroxidase (GPx; Ursini et al., 1982). As a member of the GPxs family, GPX4 can catalyze the reduction of peroxides at the expense of glutathione. However, GPX4 is unique among the GPxs in that it has a high specific affinity for large, membrane-bound substrates such as phospholipids and cholesterol hydroperoxides (Ursini et al., 1985; Maiorino et al., 1991; Sattler et al., 1994; Bao et al., 1997), and directly reduces membrane-bound phospholipid hydroperoxides *in situ* and thus protects against membrane damage. Lipid hydroperoxides have been implicated in a variety of pathophysiological processes, including inflammation, atherogenesis, neurodegeneration, and aging. Besides being the sole antioxidant enzyme able to repair lipid peroxides, GPX4 is a moonlighting protein that also regulate eicosanoid biosynthesis (Weitzel and Wendel, 1993; Schnurr et al., 1996; Huang et al., 1998; Imai et al., 1998) and cytokine signaling (Brigelius-Flohé, 2006). Its identification as the central regulator of ferroptosis, a newly discovered iron-dependent non-apoptotic form of cell death, is the so far latest addition to the list of surprises (Yang et al., 2014; Yang and Stockwell, 2016).

**FIGURE 1:**
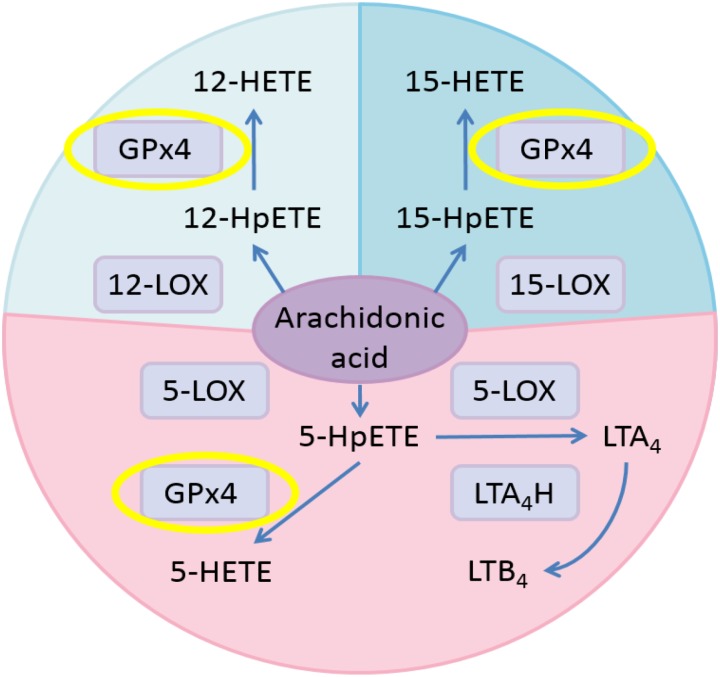
Eicosanoid biosynthesis pathways related to GPX4 in the AA metabolic network. Pink, 5-LOX pathway; pale blue, 12-LOX pathway; blue, 15-LOX pathway.

Hydroperoxides relevant to inflammation are those formed from arachidonic acid (AA) by stereoselective lipoxygenases. In human cells, AA is metabolized into pro-inflammatory leukotrienes (LTs) through the pathway involving 5-lipoxygenase (5-LOX) and leukotriene A4 hydrolase (LTA_4_H; Buczynski et al., 2009; **Figure 1**). Meanwhile, AA is oxidized to 5-HpETE, 12-HpETE, and 15-HpETE by 5-LOX, 12-lipoxygenase (12-LOX), and 15-lipoxygenase (15-LOX), respectively. Subsequently, GPX4 catalyzes the reduction of HpETEs to generate the corresponding HETEs. Pro-oxidative lipoxygenases and anti-oxidative GPxs are key regulators of systemic redox homeostasis. Previous research results suggested that activating GPX4 is beneficial to suppress inflammation (Yang et al., 2007, 2008; Pei et al., 2014; Meng et al., 2015).

Therefore, it becomes highly desirable to identify small molecules able to activate GPX4. However, compared to small molecule inhibitors, the discovery of small molecule activators is much more challenging (Zorn and Wells, 2010). In our previous study, we have identified human 15-LOX activators and demonstrated their potential in inflammation control (Meng et al., 2016). Recently, we reported the discovery of one series of GPX4 allosteric activator compounds (Li et al., 2018) by using a combining approach of potential binding cavity identification (Yuan et al., 2011, 2013), allosteric site prediction (Ma et al., 2016), and allosteric ligand virtual screen. In the present study, we identified a new compound with different chemical scaffold that can activate the enzyme activity of GPX4 by more than two folds. This compound can activate GPX4 in cell-free assay and suppress the generation of pro-inflammatory lipid mediators in cellular as well as *ex vivo* assays.

## Materials and Methods

### Reagents and Cell Culture

The compound selected from virtual screen was purchased from SPECS with purity of more than 95% (confirmed by SPECS with NMR and LC-MS; data are available on the website http://www.specs.net/). Reagents were purchased from Sigma–Aldrich unless otherwise noted. The pQE-30 bacterial expression plasmid of the U46C mutant of human cytosolic GPX4 (c-GPX4) was a generous gift from Professor Hartmut Kuhn (University Medicine Berlin-Charité, Germany). IPTG, PMSF, DTT, EDTA, and glutathione were from Amresco. Standard compounds for LC-MS/MS were purchased from Cayman Chemical. Calcium ionophore A23187 was obtained from J&K Chemical. Zileuton was purchased from Tocris Bioscience. The Symmetry C18 reverse-phase column (3.5 μm, 2.1 mm × 150 mm) was purchased from Waters Corp. HEK293T cells were received as a gift from Professor Jincai Luo (Peking University, China). The Dual-Glo Luciferase Assay System was from Promega. The pCDNA3.1 plasmid containing the gene encoding firefly luciferase was a generous gift from Professor Peng Chen (Peking University, China). Antibody against GPX4 was obtained from Abcam (no. ab125066). HT-1080 cells were the generous gift of Professor Chu Wang (Peking University, China).

### Molecular Docking Studies

The details of allosteric site prediction and virtual screen are described in a recent report (Li et al., 2018). Potential allosteric site in GPX4 was identified using the CAVITY (Yuan et al., 2011, 2013) and CorrSite (Ma et al., 2016) program, and then applied to screen for potential allosteric activator. Rigid body docking was first performed with default parameters using the program Glide SP mode (Friesner et al., 2004; Halgren et al., 2004) to screen the SPECS library (May 2013 version for 10 mg; 197,276 compounds). Flexible ligands and rigid receptor docking was then performed using the program Glide XP mode with default parameters to further screen the top 10,000 compounds from the SP results. At last, the binding conformations of the top 2,000 compounds from Glide XP were exported, including the binding conformation of compound **102**.

### Protein Expression and Purification

The recombinant mutant plasmid was transformed to *Escherichia coli* strain M15. Recombinant cells were cultivated at 37°C in LB medium containing kanamycin (25 μg/mL) and ampicillin (100 μg/mL). GPX4 expression was induced by addition of IPTG (1 mM) at an OD_600_ of 0.6, and the cells were further cultured at 30°C for 5 h. Then cells were harvested by centrifugation (6,000 rpm, 15 min), resuspended in lysis buffer [Tris-HCl (100 mM, pH 8.0), NaCl (300 mM), imidazole (20 mM), TCEP (5 mM), and PMSF (1 mM)], and incubated on ice with 1 mg/mL lysozyme for 30 min. After sonication, the lysate was centrifuged (17,000 rpm, 40 min) and the supernatant was purified using a nickel-nitrilotriacetic acid column (HisTrap HP; GE Healthcare) at 4°C. The his-tagged GPX4 was eluted with an increasing imidazole step-gradient (100–250 mM). The eluted enzyme was further subject to dialysis for 12 h [Tris-HCl (100 mM, pH 7.4), DTT (1 mM), and glycerol (20% v/v)] to remove the bulk of imidazole. The final purity of the enzyme was more than 95% as confirmed by SDS-PAGE. Protein concentrations were measured using Nanodrop 2000 (Thermo Scientific, United States).

### Activation of GPX4 in a Cell-Free Assay

By coupling the oxidation of NADPH to NADP^+^ by oxidized glutathione in the presence of glutathione reductase, GPX4 activity was assessed by measuring the decrease in NADPH fluorescence emission at 460 nm (excitation at 335 nm). To evaluate the effects of compound on GPX4 activity, compound was first pre-incubated with purified GPX4 in the assay buffer [Tris-HCl (100 mM, pH 7.4), EDTA (5 mM), Triton X-100 (0.1% v/v), NADPH (0.2 mM), glutathione (3 mM), and glutathione reductase (1 unit)] for 5 min at 37°C. Then the reaction was initiated by adding *tert*-butyl hydroperoxide (25 μM). Compound was dissolved in DMSO at a final concentration of 5%, which did not perturb the assay. Fluorescence signals were recorded for 6 min on a plate reader (Synergy, Biotek). The initial reaction rates at different concentrations of compound were used to determine EC_50_ values, which were calculated with a four-parameter logistical model of log dose against percentage activation and were obtained from at least three sets of experiments. Assay system without adding GPX4 was used to exclude the influence of the redox properties of compound. Control experiments were also conducted to confirm that the compound was not glutathione reductase activator.

Mutagenesis experiments were carried out according to the instructions of the QuikChange Site-Directed Mutagenesis (SBS Genetech Co., Ltd, Beijing, China). The pQE-30 bacterial expression plasmid of the U46C mutant of human c-GPX4 was mutated to obtain the D23A mutant. The DNA sequence of the D23A mutant was verified by DNA sequencing. The protein expression and activity assays of the D23A mutant were performed as described for the U46C mutant.

### Influence of the GPX4 Activator in Human Polymorphonuclear Leucocytes (PMN)

Following the reported procedure (Yang et al., 2007), human PMNs were isolated from the venous blood of healthy volunteers who had not ingested any aspirin-like compounds in the preceding two weeks, and then diluted to the concentration of 1 × 10^6^ cells/mL using PBS buffer. The purity of isolated PMNs was more than 95% (confirmed by Wright staining), and the concentration of PMNs was determined by trypan blue staining; 300 μL diluted PMNs were pre-incubated with vehicle (DMSO), Zileuton (an inhibitor of 5-LOX), and GPX4 activator at 37°C for 15 min, respectively. To stimulate the PMNs, 10 μM A23187, 2 mM CaCl_2_, and 500 μM MgCl_2_ were added. After different incubation periods (5–120 min) at 37°C, reactions were terminated by the addition of cold methanol (700 μL of methanol was added into 300 μL of reaction solution). 15(S)-HETE-d_8_ and PGB_2_ were added as internal standards. The upper solvent was evaporated under a stream of nitrogen, and the residue was dissolved in 150 μL of methanol before LC-MS analysis.

### LC-MS/MS Method

LC separation was performed with a Shimadzu Prominence UFLC XR (Shimadzu Corporation). The multiple-reaction-monitoring (MRM) spectra were obtained with a QTRAP 5500 mass spectrometer (AB SCIEX) equipped with an ESI source. A Waters Symmetry reverse-phase C18 column (2.1 mm × 150 mm, 3.5 μm) was used for the LC separation as described (Meng et al., 2016). Up to 35 eicosanoids and two internal standards [15(S)-HETE-d_8_ and PGB_2_] were monitored. Optimized LC-MS/MS parameters for the analysis of eicosanoids are in accordance with previous research (Meng et al., 2016).

### Inhibition of Cyclooxygenase 1 (COX-1) and Cyclooxygenase 2 (COX-2) in Cell-Free Assays

Cyclooxygenase (COXs) can convert AA to PGG_2_, and further reduce PGG_2_ to PGH_2_. The enzyme activities of COXs were determined spectrophotometrically by monitoring the oxidation of *N,N,N’,N’*-tetramethyl-*p*-phenylenediamine (TMPD) at 610 nm during the conversion of PGG_2_ to PGH_2_. The test compound was pre-incubated with purified human recombinant cyclooxygenase 1 (COX-1) or cyclooxygenase 2 (COX-2) (Cayman Chemical) for 15 min. Next, TMPD solution was added, and the reaction was initiated by adding a solution of AA. Absorbance at 610 nm was recorded on a plate reader (Synergy, BioTek). Inhibition activities were measured as described (Chen et al., 2011).

### Inhibition of Microsomal Prostaglandin E_2_ Synthase 1 (mPGES-1) in a Cell-Free Assay

The enzyme activity of human recombinant microsomal prostaglandin E_2_ synthase 1 (mPGES-1) was measured by assessment of PGH_2_ conversion to PGE_2_ (He et al., 2013). The test compound was pre-incubated with enzyme sample for 15 min. Next, PGH_2_ was added to start the reaction. After 1 min, stop solution was added to terminate the reaction. Production of PGE_2_ in the reaction mixture was measured with the PGE_2_ EIA kit (Cayman Chemical). Inhibition was measured in a similar manner as that of GPX4.

### Inhibition of 5-LOX in a Cell-Free Assay

The enzyme activity of human recombinant 5-LOX was determined spectrophotometrically by monitoring the oxidation of H_2_DCFDA to the highly fluorescent 2’,7’-dichlorofluorescein during the catalytic reaction mediated by 5-LOX as reported (Pufahl et al., 2007). The test compound was pre-incubated with enzyme for 10 min. Next, the reaction was initiated by the addition of AA, and fluorescence signals (excitation at 500 nm and emission at 520 nm) were recorded on a plate reader (Synergy, BioTek). The human 5-LOX protein was prepared as previous reported (Wu et al., 2012).

### Inhibition of LTA_4_H in a Cell-Free Assay

Leukotriene A4 hydrolase activity was measured by monitoring the formation of LTB_4_ with an enzyme-linked immunosorbent assay. The test compound was pre-incubated with purified human recombinant LTA_4_H for 15 min. Next, LTA_4_ was added to initiate the reaction. After 10 min, the reaction mixture was diluted to stop the reaction, and the production of LTB_4_ was measured with the LTB_4_ ELISA kit (Cayman Chemical). Inhibition activities were measured as described (Jiang et al., 2008).

### Activation and Inhibition of 12-LOX and 15-LOX in Cell-Free Assays

The enzyme activities of 12-LOX and 15-LOX were assessed spectrophotometrically by measuring the formation of 12-HpETE and 15-HpETE, respectively. The test compound was pre-incubated with purified human recombinant 12-LOX or 15-LOX for 1 min. Next, AA was added to initiate the reaction. Absorbance of product at 235 nm was monitored on a plate reader (Synergy, BioTek). Activation or inhibition activities were measured as described (Meng et al., 2016).

### Reduction of the Intracellular ROS Level in HEK293T Cells

HEK293T cells were cultured in Dulbecco’s modified Eagle’s medium (DMEM; Gibco) supplemented with 10% fetal bovine serum (FBS; Gibco) and 1% Pen-Strep (Gibco) in a humidified atmosphere with 5% CO_2_ at 37°C. The day before the experiment, 25,000 cells/well were seeded in eight-well chambers (Thermo Scientific). The day of the experiment, the medium was changed to fresh DMEM containing different concentrations of compound **102**. After 30 min incubation at 37°C in a tissue culture incubator, TNFα (5 ng/mL) was added to stimulate the formation of ROS for 6 h. Then, the medium was changed to fresh DMEM containing DCFH-DA (10 μM) and Hoechst 33342 for ROS analysis and nuclei stain. After 1 h incubation, the intracellular ROS levels were analyzed using a confocal microscopy (Zeiss LSM 700) equipped with 488 nm laser for excitation. In addition, to quantitively analyze the intracellular ROS levels with a plate reader, similar experiments were performed in 96-well plates. The day before the experiment, HEK293T cells were seeded in 96-well plates (8,000 cells/well). The day of the experiment, the medium was changed to fresh DMEM containing different concentrations of compound **102**. After 30 min incubation at 37°C in a tissue culture incubator, TNFα (5 ng/mL) was added to stimulate the formation of ROS for 6 h. Then, the medium was changed to fresh DMEM containing DCFH-DA (10 μM) for ROS analysis. After 20 min incubation, the fluorescence signals (excitation at 488 nm and emission at 525 nm) were recorded on a plate reader (Synergy, BioTek). The intracellular ROS and lipid ROS were also quantified by flow cytometry using DCFH-DA and C11-BODIPY. The day before the experiment, 200,000 cells/well were seeded in six-well dishes. The day of the experiment, the medium was changed to fresh DMEM containing different concentrations of compound **102**. After 30 min incubation at 37°C in a tissue culture incubator, TNFα (5 ng/mL) was added to stimulate the formation of ROS for 6 h. Then cells were harvested by trypsinization, resuspended in 500 μL Hanks Balanced Salt Solution (HBSS; Gibco) containing DCFH-DA (25 μM), or C11-BODIPY (581/591; 2 μM; Invitrogen), and incubated for 10 min at 37°C in a tissue culture incubator. Cells were then resuspended in 500 μL of free HBSS, strained through a 40 μm cell strainer (BD Falcon), and analyzed using a flow cytometer (BD LSRFortessa Cell Analyzer, BD Biosciences) equipped with 488 nm laser for excitation. Data were collected from the FL1 channel. A minimum of 10,000 cells were analyzed per condition.

### Inhibition of the NF-κB Pathway by GPX4 Activator in Luciferase Activity Assay

All plasmid DNAs were prepared using the TIANprep Mini Plasmid Kit (TIANGEN). HEK293T cells were grown to 70% confluency in 96-well plates (Corning) at 37°C in DMEM supplemented with 10% FBS, and treated with Entranster^TM^-H (Engreen) transfection reagent (0.1 μL) and purified plasmids [0.25 μg pGL4.74 (hRluc/TK) and 0.25 μg pGL4.32(luc2P/NF-κB-RE/Hygro plasmid)] in 50 μL DMEM/10% FBS per well; 24 h later, GPX4 activator was incubated with the cells (2 × 10^5^ cells per well) for 30 min. After incubation, TNFα (5 ng/mL) was added to stimulate the cells for 6 h. Then the luciferase assays were carried out using the Dual-Glo Luciferase Assay System (Promega) with a BioTek synergy 4 Multi-Mode Microplate Reader. In the control group, cells without compound **102** treatment were stimulated by TNFα for 6 h. Then the solution of compound **102** was added immediately before the Dual-Glo Luciferase Assay was started.

The effects of compound **102** on luciferase activity were tested using HEK293T cells expressing wild type luciferase. A pCDNA3.1 plasmid containing the gene encoding firefly luciferase was transfected into HEK293T cells; 24 h later, cells were lyzed by 100 μL luciferase-reporter lysis buffer (Promega E1531). Compound **1** (500 μM) was added to 40 μL cell lysate in a 96-well black plate. After incubation for 10 min, 200 μL luciferin solution (containing 20 mM tricine, 1.0 mM MgSO4, 0.1 mM EDTA, 33 mM dithiothreitol, 270 μM coenzyme A, 500 μM D-luciferin, and 530 μM ATP, pH 7.8) was added, and then the firefly luminescence was measured with a BioTek synergy 4 Multi-Mode Microplate Reader.

### Cellular Thermal Shift Assay (CETSA)

HEK293T cells cultured in 100 mm × 20 mm tissue culture dishes were treated with media containing DMSO or GPX4 activator for 3 h at 37°C in a tissue culture incubator (with 5% CO_2_). After incubation, cells were harvested and washed with PBS in order to remove excess drug. Subsequently, equal amounts of cell suspensions (1.0 × 10^6^ cells per data point) were aliquoted into 0.2 mL PCR microtubes, and excess PBS was removed by centrifugation. Cells in each microtube were resuspended in 10 μL PBS, and heated at different temperatures for 3 min and cooled to 25°C for 3 min. Subsequently, 30 μL PBS containing complete protease inhibitor cocktail was added to each microtube. The cell suspensions were lyzed using liquid nitrogen and three repeated cycles of freeze-thaw. The soluble fraction (lysate) was separated from the cell debris by centrifugation at 20,000 *g* for 20 min at 4°C. The supernatants were transferred to new microtubes and analyzed by western blot. Briefly, equal amount of samples were loaded onto 12% SDS–PAGE gels, transferred to polyvinylidene difluoride (PVDF) membranes and analyzed using antibody against GPX4 at a concentration of 1:2,000. Antibody against GPX4 was obtained from Abcam (no. ab125066).

### GPX4-Specific Activity Assay

Glutathione peroxidase 4-specific activity was measured in GPX4 mouse embryonic fibroblasts (MEFs). GPX4 specific activity was carried out in a coupled assay, by monitoring the consumption of NADPH in the presence of glutathione reductase upon addition of phosphatidyl choline hydroperoxide (Roveri et al., 1994).

### Viability Assay

Cells were seeded onto 96-well plates (5,000 cells per well) and allowed to attach overnight. The next day, the cells were pre-treated for 1 h with 100 μM compound **102**. Upon that medium was changed and cells were exposed to 20 μM ChOOH. After 24 h, viability was assessed using the AquaBluer assay as previously described (Friedmann Angeli et al., 2014).

### Determination of GSH Levels

HT-1080 cells were cultured in DMEM containing 10% FBS, 1% supplemented non-essential amino acids and 1% Pen-Strep mixture (all from Gibco), and maintained in a humidified atmosphere with 5% CO_2_ at 37°C. The day before the experiment, two million cells were seeded in 10 cm dishes. The day of the experiment, cells were treated with different concentrations of erastin to reduce GSH production followed by harvesting to determine cell number. Two million live cells from each sample were transferred to new tubes and centrifuged at 1,000 rpm for 5 min at 4°C. The cell pellet was resuspended in 1 mL sodium phosphate buffer (10 mM, pH = 7) containing 1 mM EDTA, and then sonicated. The lysate was centrifuged at 13,200 rpm for 10 min at 4°C, and cleared lysate was used to determine the amount of GSH in the sample using the QuantiChrom glutathione assay kit (BioAssay Systems).

### Prevention of Erastin-Induced Ferroptosis by GPX4 Activator in HT-1080 Cells

HT-1080 cells were cultured in DMEM containing 10% FBS, 1% supplemented non-essential amino acids and 1% Pen-Strep mixture (all from Gibco) and maintained in a humidified atmosphere with 5% CO_2_ at 37°C. The day before the experiment, 2,000 cells/well were seeded in 96-well plates (Corning). The day of the experiment, the medium was changed to fresh DMEM containing different concentrations of GPX4 activator. After 30 min incubation at 37°C in a tissue culture incubator, erastin (10 μM) or RSL3 (10 nM) was added to stimulate ferroptosis. After 24 h, cell viability was determined by MTS assay according to the instructions of the CellTiter 96^®^ AQ_ueous_ One Solution Cell Proliferation Assay System (Promega). In brief, 10 μL CellTiter 96^®^ AQ_ueous_ One Solution Reagent was added to each well. Then cells were incubated at 37°C for 1–4 h in a humidified, 5% CO_2_ atmosphere. After incubation, the absorbance at 490 nm was recorded with a BioTek synergy 4 Multi-Mode Microplate Reader.

### Testing of Reducing Effects

The stable radical 2,2-diphenyl-1-picrylhydrazyl (DPPH) assay (Dixon et al., 2012) was used for the testing. The antioxidant trolox was used as the positive control.

### Iron Chelating Ability Assay

The Prussian blue assay was used for the testing. The iron chelators deferoxamine (DFO), ciclopirox olamine (CPX), and EDTA were used as the positive control; 50 μL of each test compound dissolved in DMSO (10 mM) was added to 50 μL of FeSO_4_ (3 mM) and incubated for 10 min. After incubation, 50 μL of K_3_[Fe(CN)_6_] (2 mM) was added and the absorbance at 680 nm was recorded with a BioTek synergy 4 Multi-Mode Microplate Reader.

## Results

### Identification of GPX4 Allosteric Activator

**FIGURE 2:**
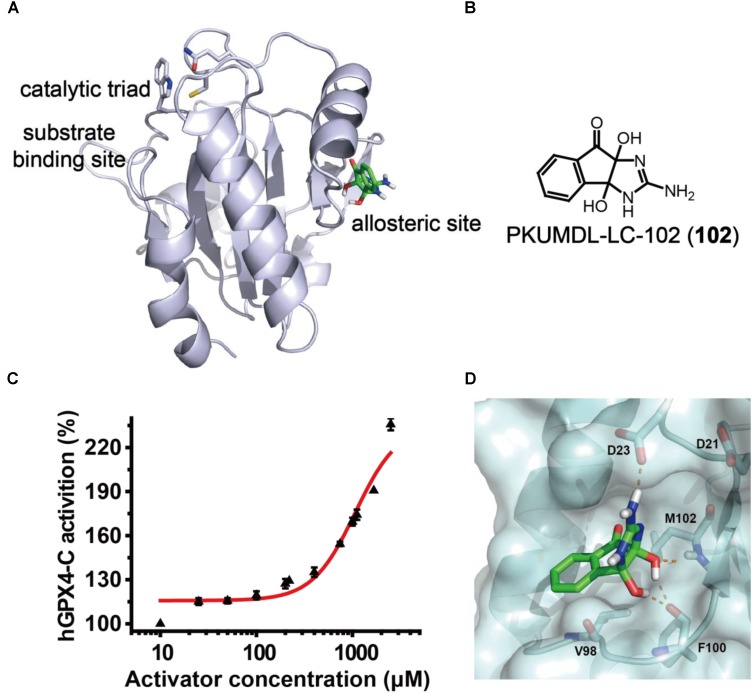
GPX4 activator identification. **(A)** The predicted allosteric site using CAVITY and CorrSite is on the opposite side from the substrate-binding site across the protein. The catalytic triad C46, Q81, and W136 in the substrate-binding site are shown as sticks. The allosteric activator is shown in green. **(B)** Chemical structure of compound **102**. **(C)** Dose–response curve for compound **102** in cell-free assay using hGPX4-C. Data shown represent the mean ± standard error of mean (SEM; *n* = 6). **(D)** Predicted binding mode of compound **102**. The hydrogen bonds between the activator (green) and hGPX4-C (pale cyan) are shown as orange dashed lines.

In the previous study, we have identified a potential allosteric site in the crystal structure of human GPX4 U46C mutant (hGPX4-C, PDB entry 2OBI; Scheerer et al., 2007) using the binding site detection program CAVITY (Yuan et al., 2011, 2013) and the allosteric site motion correlation analysis program CorrSite (Ma et al., 2016). We found a series of substituted 4-thioureidobenzenesulfonamide compounds that can activate GPX4 enzyme activity and suppress ferroptosis (Li et al., 2018). In the present study, we used a similar virtual screen procedure and the predicted allosteric site (**Figure 2A**) to screen for compounds with anti-inflammatory activity and novel chemical scaffold. Virtual screen was conducted with the SPECS compounds library (May 2013 version for 10 mg; 197,276 compounds) using molecular docking program Glide in SP and XP modes, and the activity of selected compounds was evaluated in a cell-free assay. One of the compounds, PKUMDL-LC-102 (**102**, SPECS ID AG-205/37217004; **Figure 2B**) with a new scaffold, was able to increase the enzymatic activity of hGPX4-C in a dose-dependent manner (**Figure 2C**), with maximum activation of 236%. We further demonstrated that compound **102** can increase the *T*_m_ of hGPX4-C by about 2°C (**Supplementary Figure S1**), supporting for specific binding. Compound **102** passed the PAINS (pan assay interference compounds) remover, which filters out compounds that appear as frequent hitters (promiscuous compounds) in many biochemical high throughput screens. Note that hGPX4-C is a mutant form of hGPX4 with the active site selenocysteine mutated to cysteine (U46C), resulting in much lowered enzyme activity. We further tested the activity of compound **102** in cellular assays in which GPX4 is in its native form.

The binding mode of compound **102** was predicted by molecular docking studies (**Figure 2D**). The allosteric activator interacted with the side chain carboxyl group of residue D23. As such, we generated a mutant D23A at the predicted allosteric site. The activation of hGPX4-C by compound **102** were remarkably reduced for mutant D23A (the compound concentration required to reach 150% activation was higher than 2,500 μM). As D23 could potentially form hydrogen bond with the activator, the loss of activity observed in the mutant supported such a model.

### Influence on the Inflammation Related AA Network Metabolite Distribution

Previous studies have suggested that activating GPX4 is a promising strategy to suppress eicosanoid generation and inflammation (Yang et al., 2007, 2008). Therefore, we tested the effects of GPX4 activator on eicosanoid biosynthesis in a human PMN assay. According to **Figure 1**, when GPX4 is up-regulated, an increase in 12-HETE and 15-HETE at the downstream of the 12-LOX and 15-LOX pathway, respectively, is envisioned. On the other hand, in the 5-LOX pathway, GPX4 and LTA_4_H compete for the same substrate, 5-HpETE. Hence, the ratio between the concentration of 5-HETE and leukotriene B_4_ (LTB_4_) will increase if GPX4 is activated.

**FIGURE 3:**
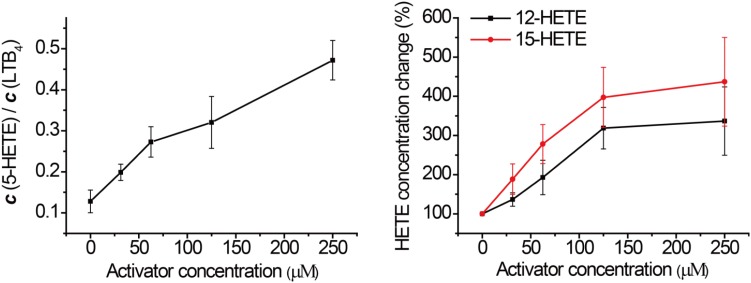
Effects of compound **102** on LOXs activities in human PMN cells. Data shown represent the mean ± standard error of mean (SEM; *n* = 3).

**FIGURE 4:**
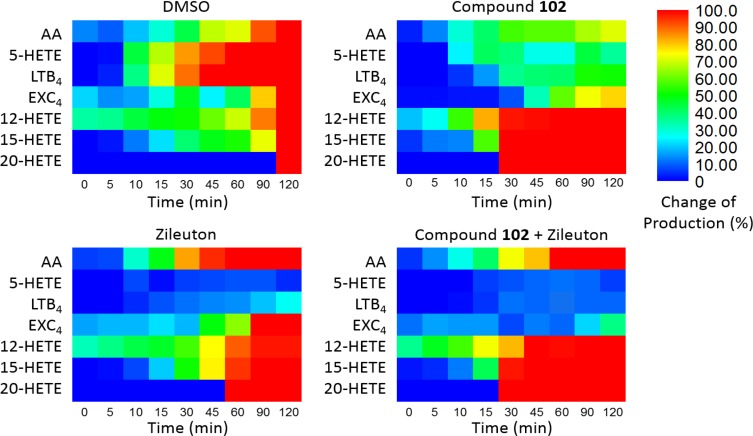
Dynamic eicosanoid production profiles in response to stimulation for 120 min. Changes in eicosanoid production of samples pre-incubated with the vehicle (DMSO), GPX4 activator **102** (125 μM), the 5-LOX inhibitor Zileuton (1 μM), and a combination of 125 μM compound **102** and 1 μM Zileuton. Change in production was defined as the normalized concentration of eicosanoid relative to that in the control set (vehicle, 120 min) multiplied by 100%. Absolute amounts of eicosanoids formed and standard error of means are given in **Supplementary Figures S4**, **S5**.

In order to assess this, human PMN cells were isolated from the venous blood of healthy volunteers who had not received non-steroidal anti-inflammatory drugs (NSAIDs) for at least 14 days. We stimulated the 5-LOX pathway using calcium ionophore A23187, and used LC-MS/MS to detect the formation of major eicosanoids derived from AA. Without GPX4 activator, the downstream metabolites of the 5-LOX, 12-LOX, and 15-LOX pathways were markedly elevated after A23187 stimulation (**Figure 4**), compared to the normal state (**Supplementary Figure S2**). Compound **102** exhibited GPX4 activating effects in a concentration-dependent manner (**Figure 3** and **Supplementary Figure S3**), with both increased ratio of 5-HETE and LTB_4_ and increased concentrations of 12-HETE and 15-HETE. The average concentration to reach 150% activation was about 39.2 μM. In addition, GPX4 has been shown to govern lipoxygenases activities in the AA metabolism (Hatzelmann and Ullrich, 1987; Hatzelmann et al., 1989; Schnurr et al., 1996; Imai et al., 1998; Sutherland et al., 2001; Chen et al., 2003; Seiler et al., 2008). The regulation of lipoxygenases by GPX4 led to a dose-dependent inhibition of the total product formation (5-HETE, LTB_4_, 12-HETE, and 15-HETE) in the presence of GPX4 activator compound **102** (**Supplementary Figure S3B**).

To verify that these phenomena were indeed caused by GPX4 activation, we tested the effects of compound **102** on other seven enzymes in the AA network, including COX-1, COX-2, mPGES-1, 5-LOX, LTA_4_H, 12-LOX, and 15-LOX. Compound **102** did not demonstrate activity against any of them in cell-free assays (less than 5% inhibition or activation at 500 μM).

### Combination of GPX4 Activator With 5-LOX Inhibitor

Furthermore, we applied compound **102** with Zileuton, a 5-LOX inhibitor clinically used as an anti-asthmatic drug with reported hepatotoxicity and neuropsychiatric side effects according to the FDA. When used alone, the inhibition of 5-LOX by Zileuton led to the accumulation of AA. Since GPX4 activator promoted the metabolism of AA through the 12-LOX and 15-LOX pathways, combination of the two should give a different distribution of AA products and reduce the accumulation of AA. To demonstrate this, we treated the stimulated PMN cells with compound **102** (125 μM) and Zileuton (1 μM). Co-administration of these two compounds attenuated the accumulation of AA and further decreased the concentrations of pro-inflammatory LTB_4_ and eoxin C_4_ (EXC_4_; **Figure 4**). Meanwhile, the concentrations of 12-HETE and 15-HETE increased, which can be further metabolized into endogenous anti-inflammatories (Meng et al., 2015). This distributary of AA caused by GPX4 activator provides a safer and more effective anti-inflammatory therapeutic strategy.

### Decrease of the Intracellular ROS Level by GPX4 Activator

**FIGURE 5:**
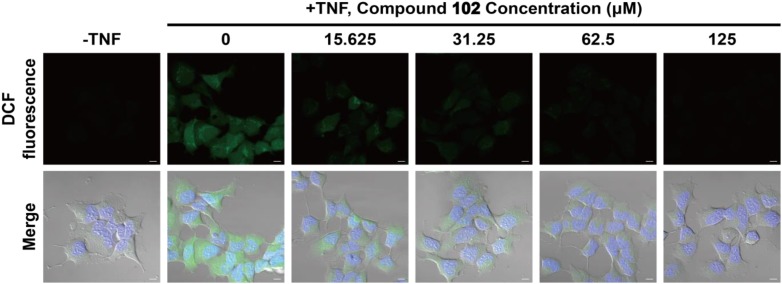
Inhibition of intracellular ROS accumulation by different concentrations of GPX4 activator in HEK293T cells. The intracellular ROS level was assayed by confocal microscopy using the fluorescent probe DCFH-DA (green). Nuclei were stained with Hoechst 33342 (blue). Scale bars represent 10 μm.

We also tested whether GPX4 activator would be effective at reducing cytosolic ROS. HEK293T cells were pre-incubated with compound **102** at increasing concentrations (15.625–125 μM). After incubation, TNFα was added to induce the formation of ROS (Blaser et al., 2016). Six hours later, the intracellular ROS level was assayed by confocal microscopy using the fluorescent probe DCFH-DA (**Figure 5** and **Supplementary Figure S6**). Compound **102** exhibited overt suppression of ROS accumulation in a dose-dependent manner with an EC_50_ of about 11.4 μM (fluorescent intensity determined by ImageJ and a plate reader, **Supplementary Figures S7**, **S8**). The intracellular and lipid ROS were also quantified by flow cytometry using DCFH-DA and C11-BODIPY. Compound **102** suppressed production of lipid ROS as well (**Supplementary Figure S9**).

### Inhibition of the NF-κB Pathway by GPX4 Activator

**FIGURE 6:**
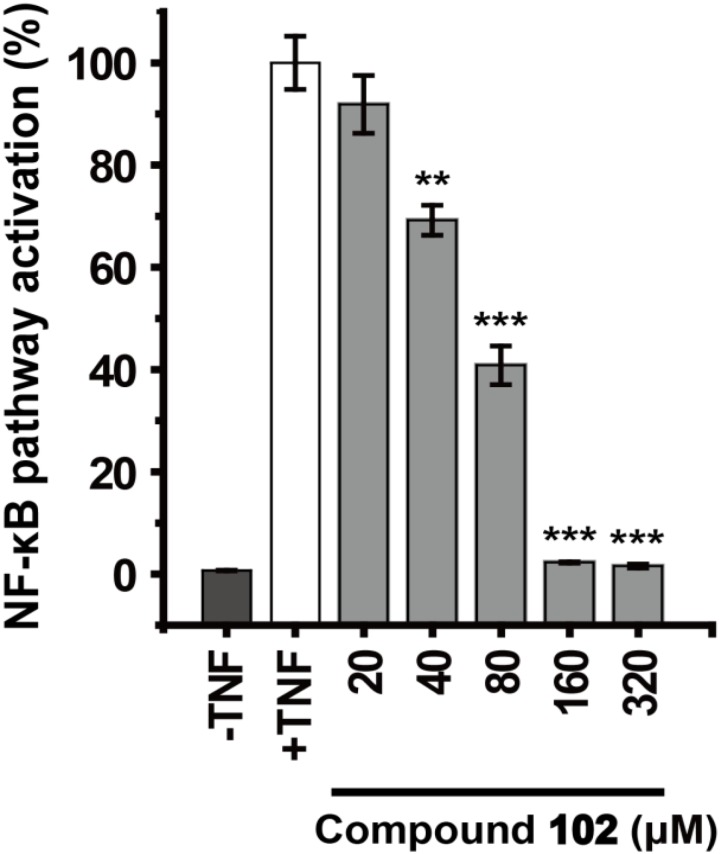
Dose-dependent inhibition of TNF induced NF-κB pathway activation by GPX4 activator. Data shown represent the mean ± standard error of mean (SEM; *n* = 5). The statistical significance was determined using a two-tailed *t*-test, ^∗∗^*p* < 0.01, ^∗∗∗^*p* < 0.001.

We further demonstrated that GPX4 activator can modulate GPX4 mediated events. GPX4 counteracts hydroperoxide-modulated events such as cytokine signaling. Direct involvement of hydrogen peroxide in the activation of NF-κB has been discussed (Morgan and Liu, 2011; Blaser et al., 2016), and GPX4 was suggested to prevent the TNF mediated activation of NF-κB (Heirman et al., 2006). We used the dual-luciferase reporter assay system to test the effects of GPX4 activator on TNF induced activation of NF-κB pathway in HEK293T cells. Compound **102** inhibited the TNF induced activation of NF-κB pathway dose-dependently with an EC_50_ of 60.8 μM (**Figure 6**). Control experiments were also performed to confirm that compound **102** would not interfere with the dual-luciferase reporter assay system (**Supplementary Figure S10**).

### Cellular Validation of the Specificity of GPX4 Activator

**FIGURE 7:**
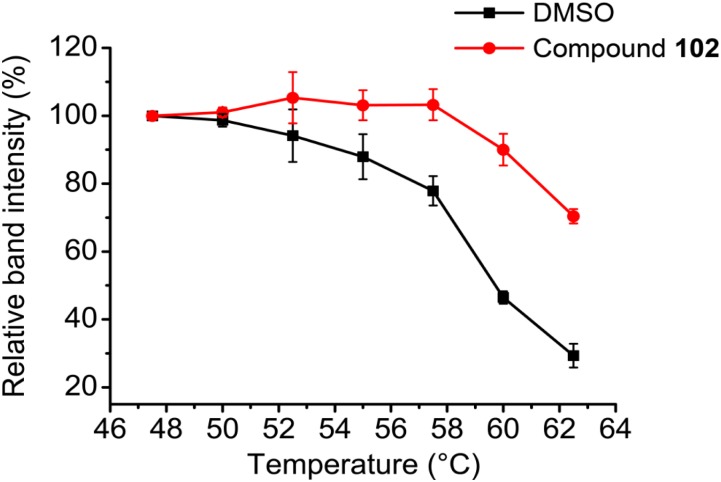
CETSA melt curves. GPX4 protein amount comparison of compound **102** (250 μM) and DMSO treated intact HEK293T cells after heated at different temperature. Data shown represent the mean ± standard error of mean (SEM; *n* = 3).

To further evaluate the selectivity and specificity of compound **102** in a cellular context, we employed the cellular thermal shift assay (CETSA; Martinez Molina et al., 2013). Western blots using antibodies against GPX4 showed that compound **102** stabilized GPX4 in intact HEK293T cells (**Figure 7** and **Supplementary Figure S11**). In addition, we measured GPX4-specific activity in MEF cell extracts, by monitoring the consumption of NADPH in the presence of glutathione reductase upon addition of phosphatidyl choline hydroperoxide. Compound **102** activated GPX4 dose-dependently (**Supplementary Figure S12A**). Furthermore, when using cholesterol hydroperoxide (ChOOH, a specific GPX4 substrate) as a stressor, compound **102** could activate GPX4 in intact cells and protect them from ChOOH toxic effects (**Supplementary Figure S12B**).

## Discussion

Despite the significant advantages to discover enzyme activators, the report of small molecule activators is rare. Using structure-based drug design methods, we successfully identified a new GPX4 allosteric activator compound. We searched SciFinder for substances with similar structures to compound **102** in related studies. Compound **102** was reported by Hong et al. (2014) as a weak inhibitor of the voltage-gated proton channel Hv1, which exhibited 20% inhibition at 200 μM. Hv1 allows sustained production of ROS by modulating NADPH oxidase (NOX) activity, and Hv1 inhibitors could be used as neuroprotective agents in ischemic stroke or anticancer drugs. We have shown that compound **102** can reduce ROS production with an EC_50_ of 11.4 μM by activating GPX4. Under 200 μM, we assume that compound **102** reduces ROS production mainly through GPX4. It would be beneficial if compound **102** can inhibit Hv1 at the same time while activating GPX4, as both lead to the reduction of ROS and neuroprotective function.

Recently, GPX4 was found to be the key player in ferroptosis regulation. Ferroptosis is negatively regulated by the enzymatic activity of GPX4, and increasing attention to possible ways of inhibiting this cell death modality are needed (Yang et al., 2014; Conrad et al., 2016). Ferroptosis is characterized by increased levels of lipid hydroperoxides and iron overload, and lipoxygenase enzymes could drive ferroptosis via increase production of 5-, 12-, and 15-HpETEs (Dixon et al., 2012; Friedmann Angeli et al., 2014; Yang et al., 2016). Suppression of ferroptosis by small molecules (e.g., ferrostatin-1 and liproxstatin-1) can protect against cell death in cellular models of Huntington’s disease, periventricular leukomalacia, kidney dysfunction, and in a pre-clinical model of ischaemia/reperfusion-induced tissue injury (Friedmann Angeli et al., 2014; Skouta et al., 2014; Hofmans et al., 2016). The ferroptosis inhibitors discovered now are not designed to take effect by specifically activating the enzyme activity of ferroptosis regulator GPX4 and accelerating the reduction of HpETEs to HETEs.

**FIGURE 8:**
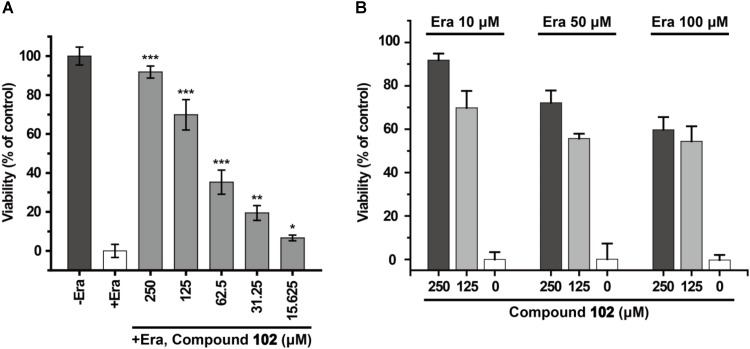
Prevention of erastin-induced ferroptosis by GPX4 activator compound **102** in HT-1080 cells. **(A)** Compound **102** prevented cell death dose-dependently with a lethal concentration of erastin (10 μM). **(B)** Death-suppression by compound **102** with increasing erastin (Era) concentrations. Data shown represent the mean ± standard error of mean (SEM; *n* = 5). The statistical significance was determined using a two-tailed *t*-test, ^∗^*p* < 0.05, ^∗∗^*p* < 0.01, ^∗∗∗^*p* < 0.001.

**FIGURE 9:**
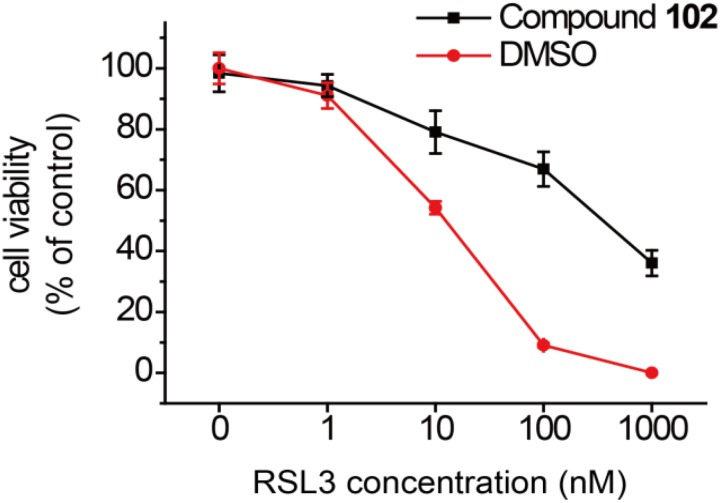
Prevention of RSL3-induced ferroptosis by GPX4 activator compound **102** in HT-1080 cells. Death suppression by compound **102** (125 μM) with increasing RSL3 concentrations. Data shown represent the mean ± standard error of mean (SEM; *n* = 5).

In order to test if our new activator could have a beneficial effect in the context of inhibiting cell death, we evaluated compound **102** for its ability to prevent erastin-induced ferroptosis. In human HT-1080 fibrosarcoma cells, treatment of erastin (Era) restricts the production of glutathione (**Supplementary Figure S13**), and therefore inactivates GPX4 and triggers ferroptosis (Yang et al., 2014). As such, HT-1080 cells were pre-incubated with GPX4 activator before treatment of erastin (10 μM). After 24 h, cell viability was determined by MTS assay. Compound **102** prevented erastin-induced ferroptosis evidently in a dose-dependent manner (**Figure 8A**), even when the concentration of erastin reached to 100 μM (**Figure 8B**). Compound **102** also suppressed ferroptosis induced by GPX4 inhibitor RSL3 (Yang et al., 2014; **Supplementary Figure S14**). Unlike the allosteric activator compound **102**, the GPX4 inhibitor RSL3 binds to the substrate binding site. Therefore, the effect of compound **102** was antagonized when the concentration of RSL3 increased (**Figure 9**). These results supported that compound **102** was a specific GPX4 activator, and increasing GPX4 activity would be an alternative way to suppress ferroptosis. We also confirmed that compound **102** was not reducing agent or iron chelator (**Supplementary Figures S15**, **S16**). Ferroptosis inhibitor acting by activating the enzyme activity of ferroptosis regulator GPX4 are more specific than lipid ROS scavenger ferrostatin-1/liproxstatins and iron chelators like DFO and CPX. GPX4 activator does not interfere with the cellular iron concentration and other iron-related physiological processes. Meanwhile, GPX4 activator enhances the overall defense of cells against oxidative damage.

## Conclusion

**FIGURE 10:**
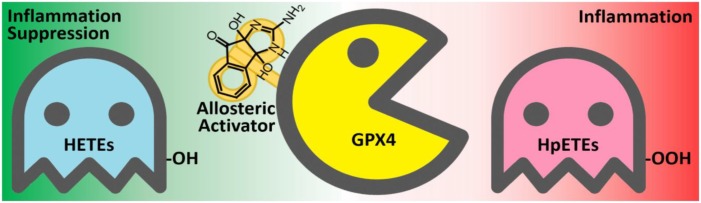
The anti-inflammatory mechanism of the GPX4 allosteric activator compound **102**. GPX4 (yellow) catalyzes the reduction of inflammation-related HpETEs (pink) to generate the corresponding HETEs (light blue). Compound **102** is an allosteric activator of GPX4 and is beneficial to inflammation suppression.

By using the GPX4 activator compound as chemical probe, we demonstrated that up-regulation of GPX4 activity can reduce the production of inflammatory agents and promote inflammation resolution (**Figure 10**). Combinations of GPX4 activator with other anti-inflammation compounds are promising inflammation intervention strategy. In addition, GPX4 activators can also be applied in a series of biological processes where GPX4 is involved, especially in ferroptosis control. Given the irreplaceable role of GPX4 in reducing lipid hydroperoxides within biological membranes, our GPX4 activator has therapeutic potential for lipid-peroxidation-related diseases such as neurodegeneration, atherogenesis, and aging. Further optimization and development of GPX4 activators are expected to provide novel solutions for treating inflammation and other lipid-peroxidation-mediated diseases.

## Author Contributions

LL and CL conceived the project and wrote the manuscript. CL performed the virtual screen, protein expression and purification, enzymatic assays, mutagenesis, and cell-based assays. XD and YL contributed to the chemical synthesis and purity analysis of the compound. XX participated in cell-free assay of GPX4 and cell experiments. JFA conducted GPX4-specific activity assay and ChOOH viability assay.

## Conflict of Interest Statement

The authors declare that the research was conducted in the absence of any commercial or financial relationships that could be construed as a potential conflict of interest.
