# Supplementary material for: Activation of Glutathione Peroxidase 4 as a Novel Anti-inflammatory Strategy
Source: Front Pharmacol. 2018 Oct 3;9:1120. doi: 10.3389/fphar.2018.01120 (PMC6178849; doi:10.3389/fphar.2018.01120)
Supplement: Supplementary file 1 [file Presentation_1.PDF]

Supplementary Material

Activation of Glutathione Peroxidase 4 as a Novel Anti-Inflammatory Strategy

Cong Li, Xiaobing Deng, Xiaowen Xie, Ying Liu, José Pedro Friedmann Angeli and Luhua Lai*

*** Correspondence:** Luhua Lai: lhlai@pku.edu.cn

1 Supplementary Figures

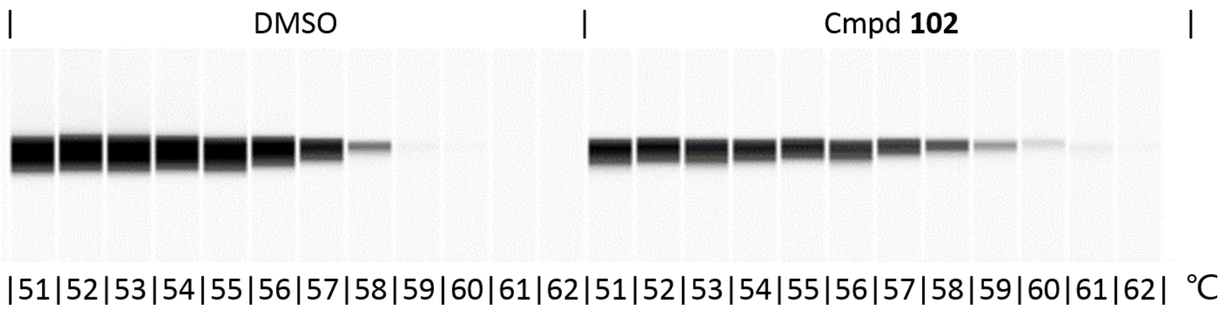

Supplementary Figure 1. Thermal shift assay western blot. GPX4 protein amount comparison of DMSO and compound **102** (cmpd **102**, 250 μM) treated purified GPX4 in the assay buffer [Tris-HCl (100 mM, pH 7.4), EDTA (5 mM), Triton X-100 (0.1 % v/v)] after heated at different temperature. Compound **102** stabilized GPX4 by about 2 ℃.

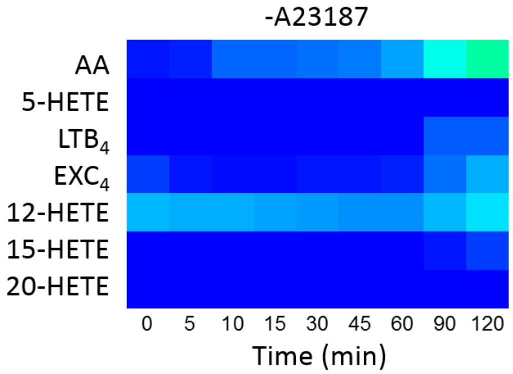

Supplementary Figure 2. Dynamic eicosanoid production profiles without stimulation for 120 min. Without GPX4 activator, the downstream metabolites of the 5-LOX, 12-LOX, and 15-LOX pathways were markedly elevated after stimulation, compared to this normal state. Change in production was defined as the normalized concentration of eicosanoid relative to that in the control set (vehicle, 120 min) and multiplied by 100 %. Absolute amounts of eicosanoids formed and standard error of means are given in Supplementary Figures S4 and S5.

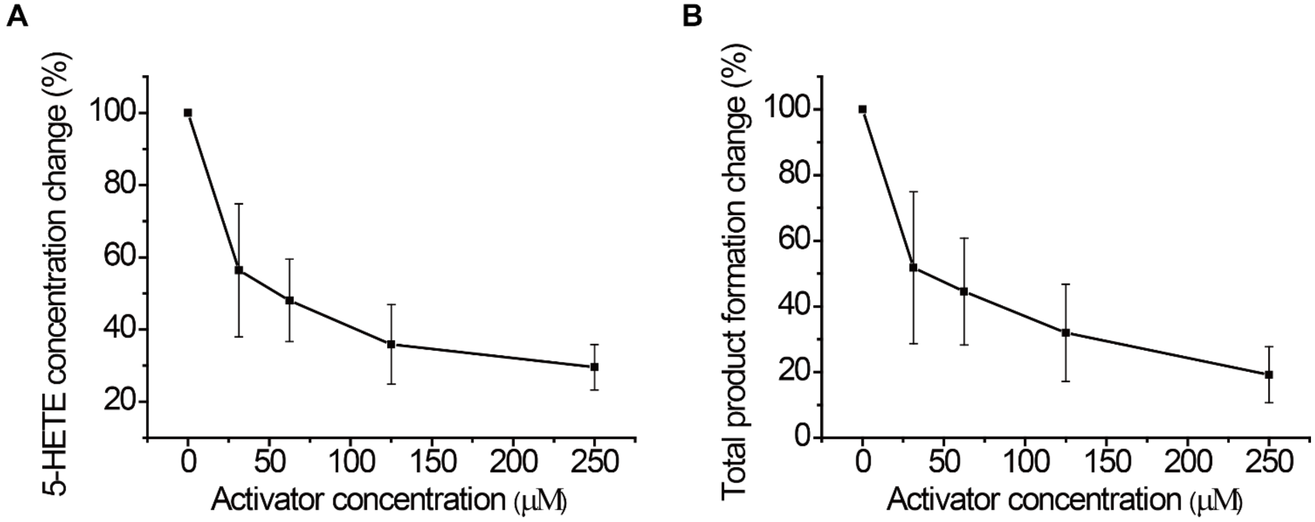

Supplementary Figure 3. Effects of compound **102** on LOXs activities in human PMN cells. **(A)** The percent change of 5-HETE concentration. **(B)** The total product formation (5-HETE, LTB_4, 12-HETE, and 15-HETE) percent change in the 5-LOX, 12-LOX, and 15-LOX pathways. Data shown represent the mean ± standard error of mean (SEM) (n = 3).

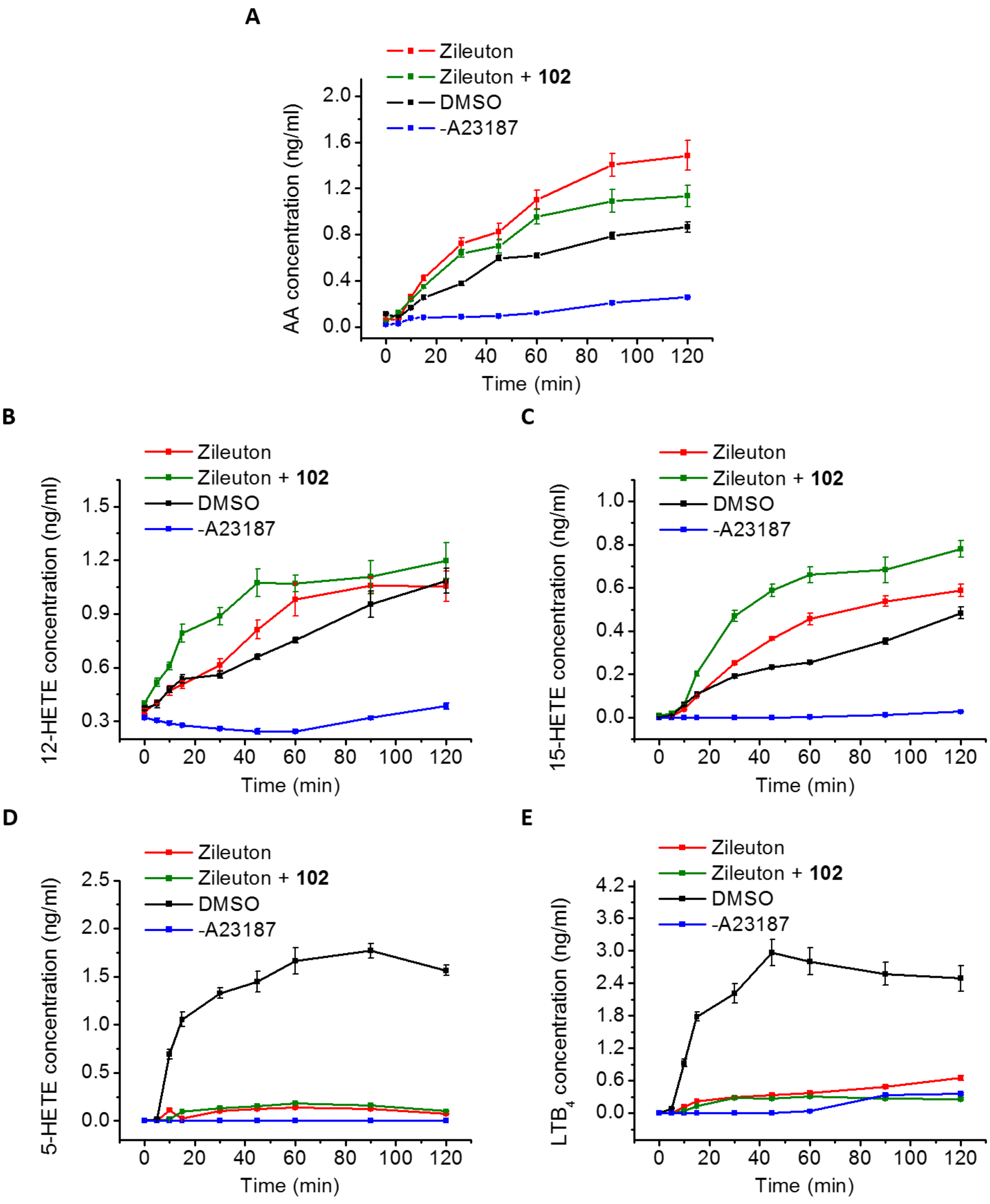

Supplementary Figure 4. Co-treatment of GPX4 activator compound **102** and 5-LOX inhibitor (Zileuton) in PMN assays. The blue lines (-A23187) represent samples without calcium ionophore A23187 stimulation, the black lines (DMSO) represent samples preincubated with the vehicle DMSO and then treated with A23187, the red lines (Zileuton) represent samples preincubated with Zileuton and then treated with A23187, and the green lines (Zileuton + **102**) represent samples preincubated with Zileuton and compound **102** and then stimulated with A23187. **(A)** Influence on the production of AA. **(B)** Influence on the production of 12-HETE. **(C)** Influence on the production of 15-HETE.

(D) Influence on the production of 5-HETE. **(E)** Influence on the production of LTB_4. Data shown represent the mean ± standard error of mean (SEM) (n = 4).

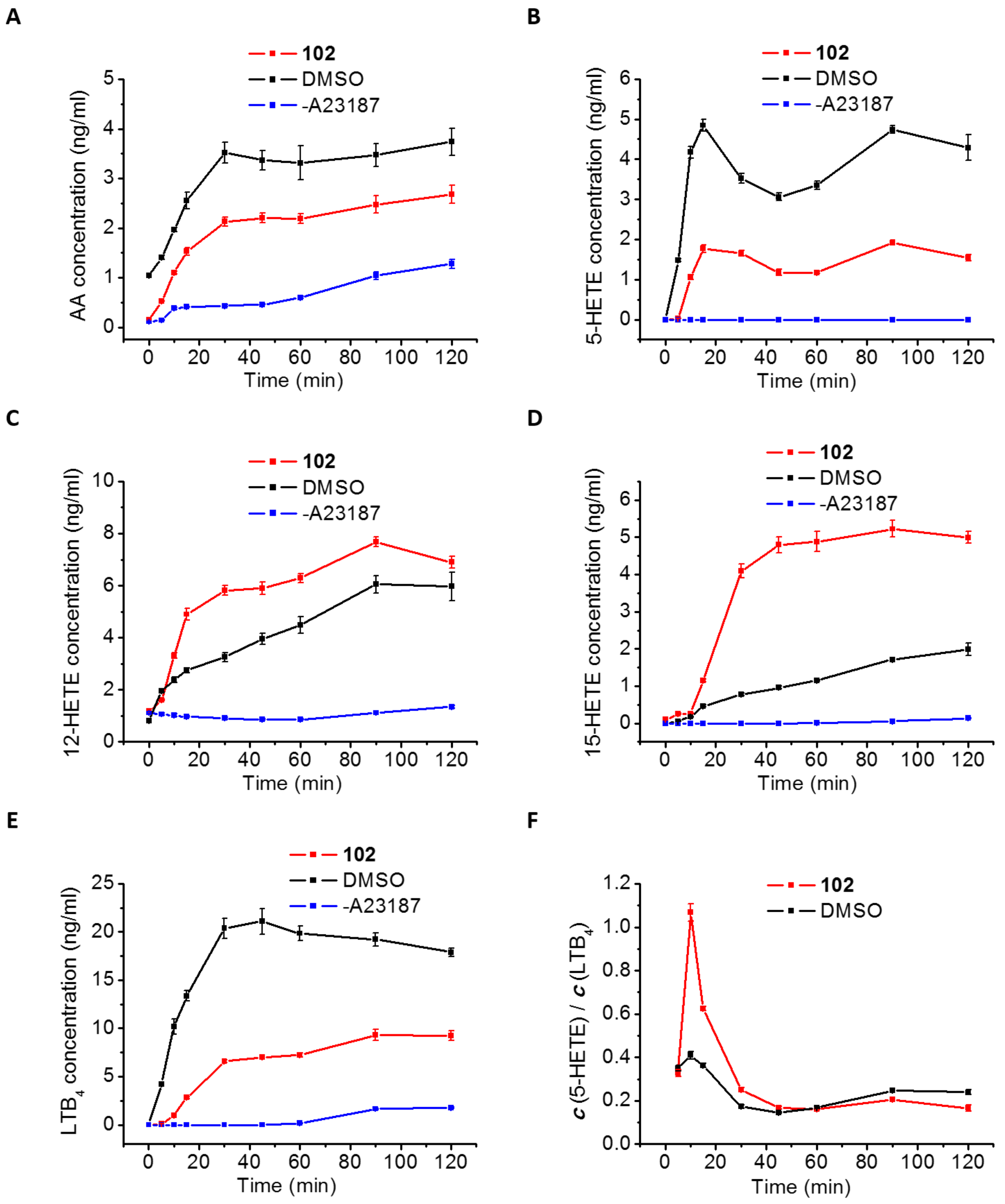

Supplementary Figure 5. Dynamic eicosanoid production profiles in PMN assays determined by LC-MS/MS. The blue lines (-A23187) represent samples without calcium ionophore A23187 stimulation, the black lines (DMSO) represent samples preincubated with the vehicle DMSO and then treated with A23187, and the red lines (**102**) represent samples preincubated with compound **102** and then stimulated with A23187. **(A)** Production of AA. **(B)** Production of 5-HETE. **(C)** Production of 12-HETE. **(D)** Production of 15-HETE. **(E)** Production of LTB_4. **(F)** The ratio between 5-HETE and LTB_4. Data shown represent the mean ± standard error of mean (SEM) (n = 4).

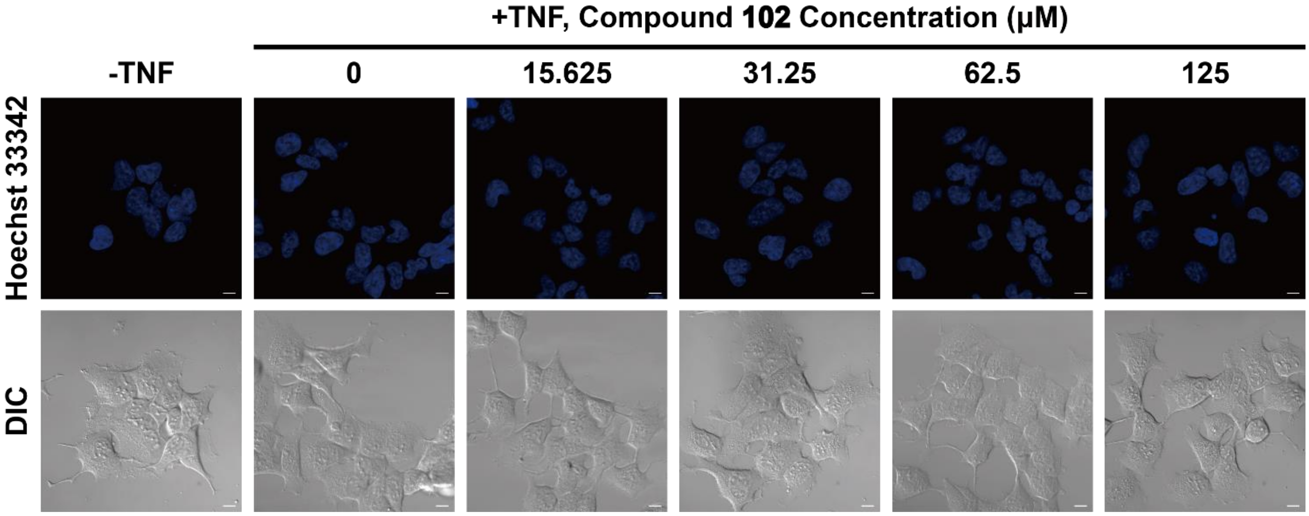

Supplementary Figure 6. Inhibition of intracellular ROS accumulation by different concentrations of GPX4 activator in HEK293T cells. Nuclei were stained with Hoechst 33342 (blue). Scale bars represent 10 μm.

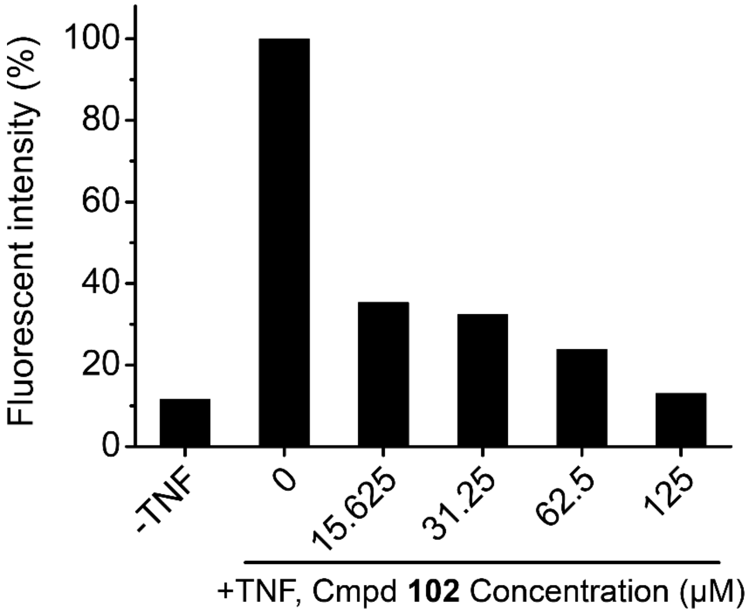

Supplementary Figure 7. Inhibition of intracellular ROS accumulation by different concentrations of GPX4 activator compound **102** (cmpd **102**) in HEK293T cells. The intracellular ROS level was assayed by confocal microscopy using the fluorescent probe DCFH-DA, and the fluorescent intensity was determined by ImageJ.

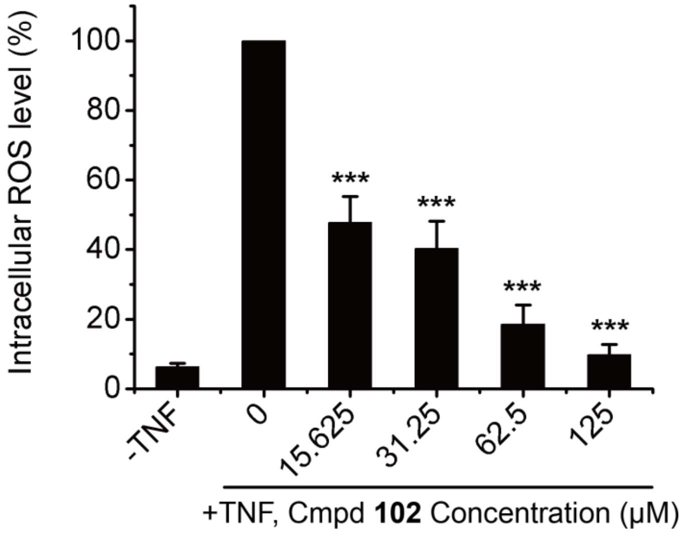

Supplementary Figure 8. Inhibition of intracellular ROS accumulation by different concentrations of GPX4 activator compound **102** (cmpd **102**) in HEK293T cells. The intracellular ROS level was assayed using the fluorescent probe DCFH-DA. Data shown represent the mean ± standard error of mean (SEM) (n = 6). The statistical significance was determined using a two-tailed t-test, ***p < 0.001.

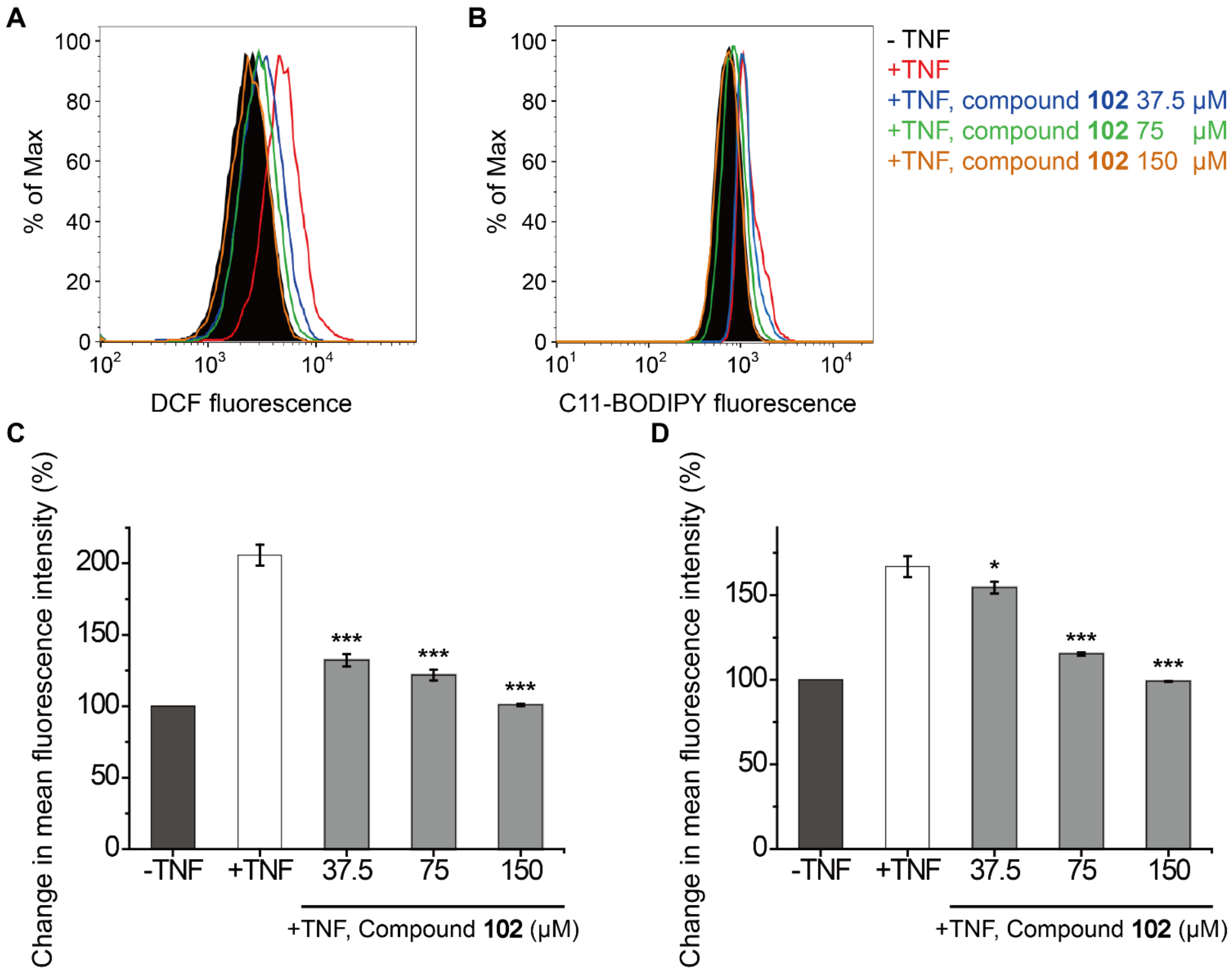

Supplementary Figure 9. Inhibition of intracellular and lipid ROS accumulation by different concentrations of GPX4 activator compound **102** in HEK293T cells. **(A)** The intracellular ROS production was assayed by flow cytometry using the fluorescent probe DCFH-DA. **(B)** The lipid ROS production was assayed by flow cytometry using C11-BODIPY. Representative data from one of three experiments are shown. **(C)** Change in intracellular ROS mean fluorescence intensity measured by flow cytometry. **(D)** Change in lipid ROS mean fluorescence intensity measured by flow cytometry. Data shown represent the mean ± standard error of mean (SEM) (n = 3). The statistical significance was determined using a two-tailed t-test, $*p < 0.05$, $***p < 0.001$.

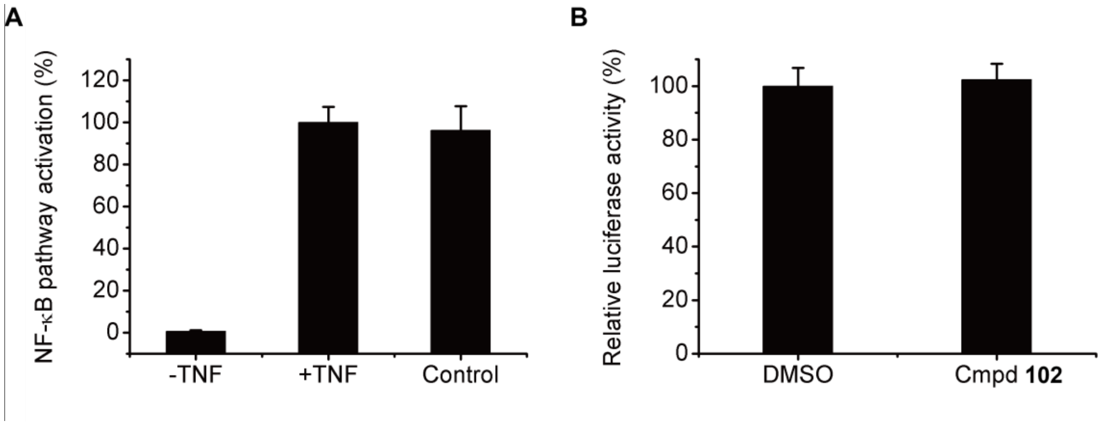

Supplementary Figure 10. Control experiments testing the effects of compound **102** (cmpd **102**) on the luciferase activity. **(A)** In the control group, cells without compound **102** treatment were stimulated by TNF for 6 h. Then the solution of compound **102** (500 μM) was added immediately before the Dual-Glo Luciferase Assay was started. **(B)** Luciferase activity assay using HEK293T cells expressing wild type luciferase. Data shown represent the mean ± standard error of mean (SEM) (n = 5).

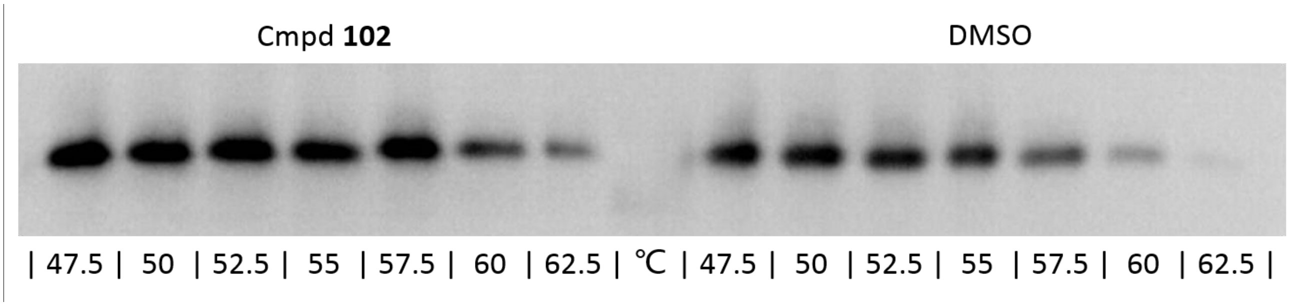

Supplementary Figure 11. Representative CETSA western blots. GPX4 protein amount comparison of compound **102** (cmpd **102**, 250 μM) and DMSO treated intact HEK293T cells after heated at different temperature.

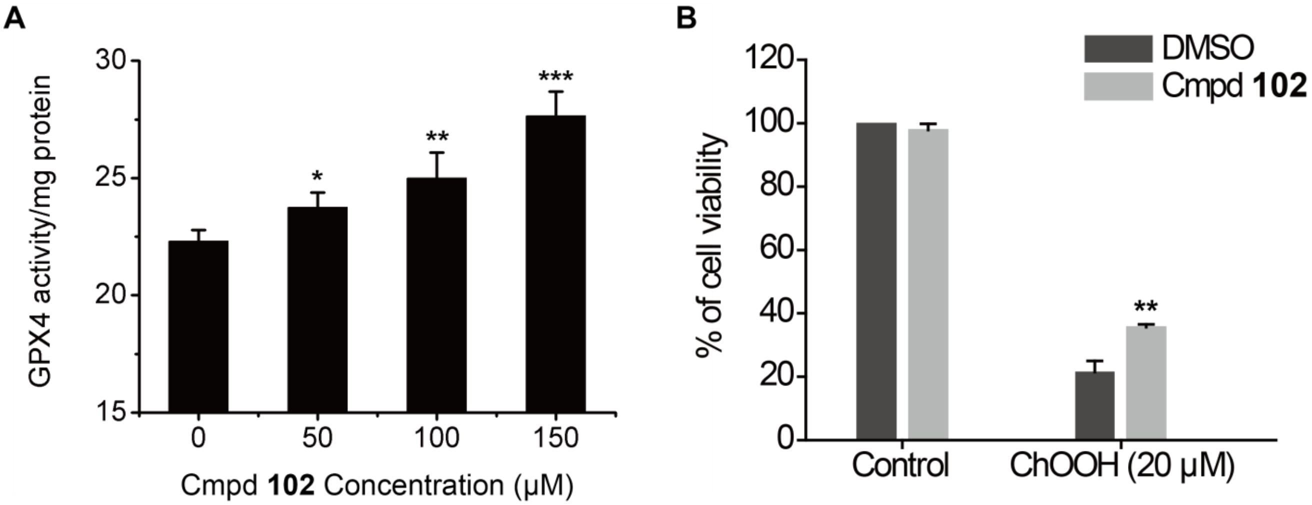

Supplementary Figure 12. Assessment of the GPX4-specific activity. **(A)** Dose-dependent GPX4 activity assay of compound **102** (cmpd **102**). GPX4 specific activity is expressed as NADPH (nmoles) consumed per mg of protein per min. Data shown represent the mean ± standard deviation (s.d.) (n = 3). **(B)** Cholesterol hydroperoxide (ChOOH) induced cell death assay. Compound **102** (100 µM) prevented cell death induced by the GPX4 specific substrate ChOOH (20 µM). Data shown represent the mean ± standard deviation (s.d.) (n = 4). The statistical significance was determined using a two-tailed t-test, *p < 0.05, **p < 0.01, ***p < 0.001.

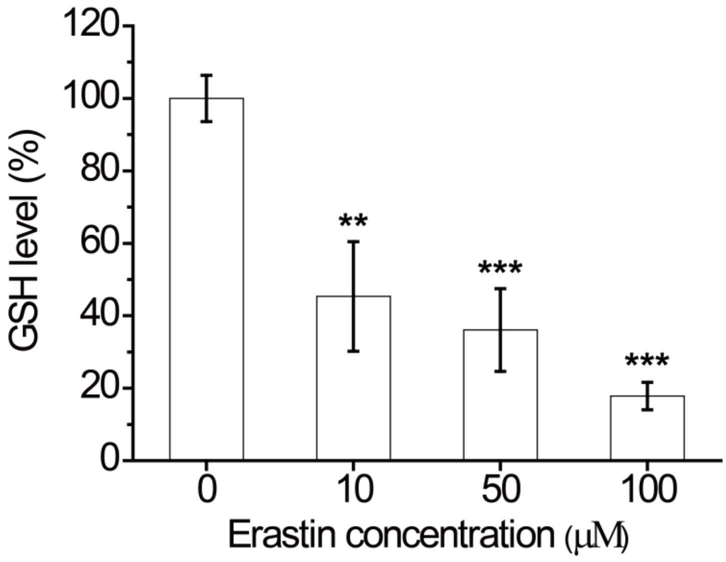

Supplementary Figure 13. Erastin restricts the production of glutathione dose-dependently in HT-1080 cells. Data shown represent the mean ± standard error of mean (SEM) (n = 5). The statistical significance was determined using a two-tailed t-test, **p < 0.01, ***p < 0.001.

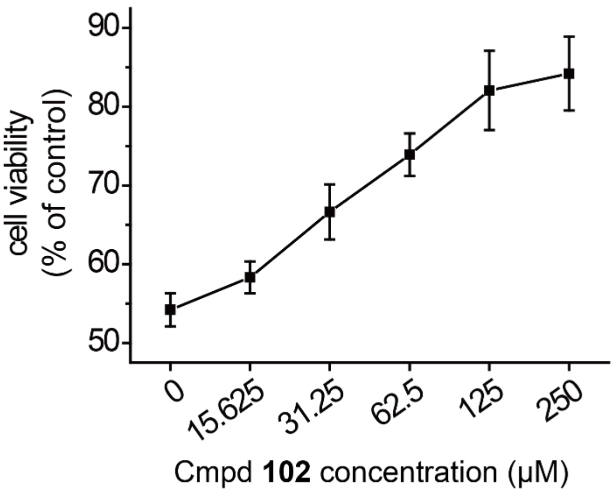

Supplementary Figure 14. Prevention of RSL3-induced ferroptosis by GPX4 activator compound **102** (cmpd **102**) in HT-1080 cells. Compound **102** prevented ferroptosis (10 nM RSL3) dose-dependently. Data shown represent the mean ± standard error of mean (SEM) (n = 5).

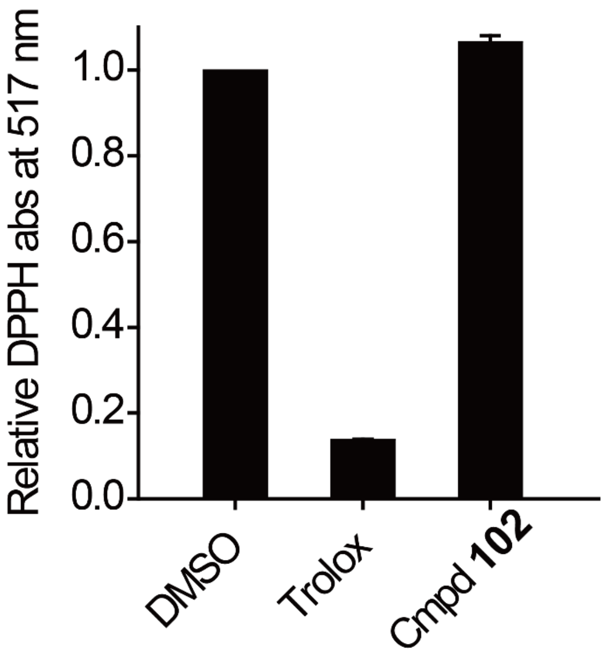

Supplementary Figure 15. Cell-free antioxidant potential monitored by changes in the absorbance at 517 nm of the stable radical DPPH. The final concentration of each test compound was 500 μM. Data shown represent the mean ± standard deviation (s.d.) (n = 3).

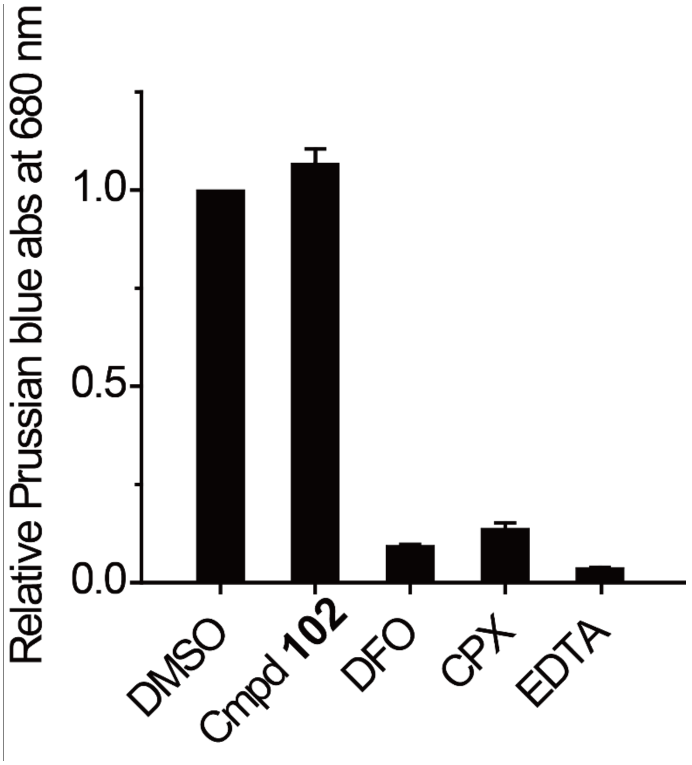

Supplementary Figure 16. Iron chelating ability monitored by the absorbance at 680 nm of the Prussian blue. The iron chelators deferoxamine (DFO), ciclopirox olamine (CPX), and EDTA were used as the positive control. Data shown represent the mean ± standard deviation (s.d.) (n = 3).
